# Recent Advances in Preclinical Research Using PAMAM Dendrimers for Cancer Gene Therapy

**DOI:** 10.3390/ijms22062912

**Published:** 2021-03-13

**Authors:** Piotr Tarach, Anna Janaszewska

**Affiliations:** Department of General Biophysics, Faculty of Biology and Environmental Protection, University of Lodz, Pomorska 141/143, 90-236 Lodz, Poland; anna.janaszewska@biol.uni.lodz.pl

**Keywords:** PAMAM dendrimer, nanoparticles, cancer, gene therapy, gene delivery, nucleic acids, RNA delivery, small and large DNA, delivery systems, preclinical research

## Abstract

Carriers of genetic material are divided into vectors of viral and non-viral origin. Viral carriers are already successfully used in experimental gene therapies, but despite advantages such as their high transfection efficiency and the wide knowledge of their practical potential, the remaining disadvantages, namely, their low capacity and complex manufacturing process, based on biological systems, are major limitations prior to their broad implementation in the clinical setting. The application of non-viral carriers in gene therapy is one of the available approaches. Poly(amidoamine) (PAMAM) dendrimers are repetitively branched, three-dimensional molecules, made of amide and amine subunits, possessing unique physiochemical properties. Surface and internal modifications improve their physicochemical properties, enabling the increase in cellular specificity and transfection efficiency and a reduction in cytotoxicity toward healthy cells. During the last 10 years of research on PAMAM dendrimers, three modification strategies have commonly been used: (1) surface modification with functional groups; (2) hybrid vector formation; (3) creation of supramolecular self-assemblies. This review describes and summarizes recent studies exploring the development of PAMAM dendrimers in anticancer gene therapies, evaluating the advantages and disadvantages of the modification approaches and the nanomedicine regulatory issues preventing their translation into the clinical setting, and highlighting important areas for further development and possible steps that seem promising in terms of development of PAMAM as a carrier of genetic material.

## 1. Introduction

Carriers used to introduce genetic material into the cells of the body are divided into viral and non-viral vectors. Over the last two decades, an ideal carrier of genetic material has been sought, which would effectively deliver genetic material inside the cell, while maintaining its functionality, without inducing unintended effects in the cell itself, such as viral insertion mutagenesis induced by the integration of viral genetic material into the host genome, leading to a disturbance of cell proliferation and an increased risk of oncogenesis [1].

Biological carriers of genetic material, such as viral vectors, are successfully used in experimental gene therapies to cure immunodeficiencies, or in the design of Chimeric Antigen Receptor T cell (CAR-T) receptors to eliminate cancer cells, where conventional immunomodulating therapies have failed to bring satisfactory results [2]. However, the use of viral vectors in gene therapies is limited, due to difficult large-scale production as well as a certain degree of immunogenicity and carcinogenicity and, therefore, current scientific efforts are directed towards the study of a wide range of molecules, such as dendrimers, which are capable of transferring genetic material into the cells [1,3]. Regardless, viral vectors are widely used because of their high tolerability, standardized transfection procedures, and more stable and robust expression compared to non-viral carriers [4].

### 1.1. Gene Therapy Challenges

In recent years, there has been a significant increase in the number of clinical trials using non-viral carriers of genetic material and drugs [5]. In spite of the wide availability of genetic material types with potential clinical use, there are currently only 20 clinically proven and commercially available gene therapies [6]. Since 1989, over 2600 clinical trials have been carried out. The USA is the biggest clinical trial contributor, with more than 1600 currently conducted studies. Other contributing countries are the UK (*n* = 221), Germany (*n* = 92) and China (*n* = 84). The main areas of research are cancer (65%) and monogenic diseases (11.1%), constituting nearly 2/3 of all currently conducted gene therapy studies. The most commonly used carriers of genetic material are adenovirus (20.5%), retrovirus (17.9%), naked/plasmid DNA (16.6%), and adeno-associated virus (7.6%). Despite promising results in the in vitro and in vivo phases, only 56.8% of all clinical the studies have reached stage I, whereby only 3.8% of these studies have successfully reached the end of stage III [7]. The most promising research fields include CAR-T-receptor studies in anticancer therapies and monogenic diseases. Their remarkable efficacy in the treatment of leukemia and lymphoma has led to the development and introduction of several CAR-T-based drugs into the therapeutic market, namely Kymriah, Yescarta, Tecartus in the EU and US, and Breyanzi in the US [8,9,10,11,12,13,14]. For other disease entities, equally promising results have been achieved using lentivirus and gamma-retrovirus carriers and ex vivo stem-cell-based therapies. In 2016, the European Marketing Authorization (EMA) approved the first ex vivo stem cell gene therapy under the name Strimvelis™ (GlaxoSmithKline) [15]. The treatment is addressed to patients affected by a very rare genetic disease called severe combined immunodeficiency due to Adenosine deaminase deficiency (ADA-SCID). A clinical trial testing the effectiveness of a given therapeutic approach involved 40 patients with a given disease, where over 70% were completely cured after the application of a given therapy [16]. Lentiviral vectors have been successfully used in clinical settings to treat haemoglobinopathies, including sickle cell disease and β-thalassemia [17,18] and the lentiviral vector-based product “Libmeldy” for the ex vivo gene therapy of children with metachromatic leukodystrophy [19,20]. In relation to the use of adeno-associated virus (AAV) in clinical trials, remarkable results have been reported in the treatment of spinal muscular atrophy with adeno-associated viral vector-based gene therapy using Zolgensma, which ultimately made it possible to stop the progression of the disease and, with appropriate specialized care and physiotherapy, led to a significant improvement in the patient’s quality of life [21]. Other approved gene therapies include Luxturna, a treatment for RPE65-mediated inherited retinal dystrophy [22,23], and Glybera, for the treatment of familial lipoprotein lipase (LPL) deficiency (LPLD) [24]. Roctavian (BMN 270), a drug designed to treat hemophilia A, is awaiting approval from the Food and Drug Administration. [25,26].

One of the main hopes of modern medicine is the potential to cure disorders where standard treatment methods provide unsatisfactory results. Although gene therapy is still a growing medical field and the technology is still in its infancy, it is nevertheless one of the tools that could potentially meet this expectation. The main challenge facing modern medicine the search for a safe and effective carrier of genetic material that is non-toxic to the patient’s cells and delivers the genetic material to the patient’s cells intact. Due to the incomplete safety of bio-based carriers, the use of synthetic carriers has been intensively developed in recent years, with noticeable success [27].

### 1.2. Viral and Non-Viral Vectors in Gene Therapies

Gene therapy is a set of techniques used to modify the patient’s phenotype for therapeutic purposes, by replacing an abnormal or non-functional gene with its normal copy [28]. It is used to treat a specific genetic disease by delivering genetic material inside the target cells. There are two strategies used to achieve this goal: ex vivo and in vivo gene transfer. The ex vivo strategy is based on the delivery of genetic material to an organ outside the patient’s body, followed by the transplantation of such modified tissue into the recipient body. The in vivo approach relies on the direct delivery of genetic material to the patient’s target tissue. The in vivo strategy can be divided depending on the need to produce or silence a specific protein in the body. In the first case, a plasmid DNA-containing therapeutic gene is delivered into the cell. Depending on the type of DNA vector and whether there is a need for continuous or transient expression, plasmid DNA is inserted into the selected location of the human genome and transcribed into the mRNA strand. In the case of some retro- and lentiviral vectors, DNA is permanently inserted into the host genome, leading to the long-term expression of the selected gene. However, in the case of liposomal and episomal vectors, the expression is only transient, which means that a given protein is produced until the cell completely degrades the given genetic material with each subsequent cell division. Generally, this strategy is aimed towards the expression of an mRNA transcript and production of a specific therapeutic protein. The efficiency of the naked antisense oligonucleotides delivery process is lower compared to nano-systems such as dendriplexes. This is caused by the rapid degradation of naked genetic material by serum nucleases and potential activation of the immune response. Moreover, the low stability of antisense oligonucleotides, resulting from nucleolytic degradation, poor efficiency of delivery process to target tissues and cells, insufficient specificity to target sequences, risk of off-targeting and cytotoxic effects, are the main obstacles preventing their use in gene therapies [29]. Hence, in order to effectively and safely transport RNA to the cell, it is necessary to use relevant carriers of genetic material [30,31,32].

Depending on the desired therapeutic effect (expression or silencing of a specific gene), different types of genetic material are used. Considering the types of nucleic acids used in cellular transport research, one can distinguish plasmids, mRNAs, antisense oligonucleotides (ASOs), short regulatory RNAs (siRNAs, miRNAs, etc.), RNA- DNA- and MNAzymes (multiple strands). After the genetic material is delivered inside the cell, utilizing existing cellular machinery, it evokes a specific biological effect. The main site of activity of mRNAs, ASOs, short regulatory RNAs and DNA-, RNA- and MNAzymes is cytosol and the nucleus, whereas the main site of plasmid activity is the nucleus. Plasmids and mRNAs are mainly used to induce the expression of a specific therapeutic protein. The main difference between the genetic material contained in plasmids and mRNAs is that plasmids may also contain information about encoded regulatory RNAs, whereas mRNAs carry information solely about the primary protein sequence. The main purpose of ASOs is the degradation of mRNA by RNAase H and/or inhibition of the translation process caused by steric hindrance. In contrast, short regulatory RNAs may lead to sequence-specific gene silencing as a result of RNA and DNA degradation, translation repression and chromatin rearrangement [33,34]. Generally, antisense technology is used to downregulate the expression of proteins at the level of mRNA that causes a specific disease entity. DNA-, RNA- and MNAzymes, as distinct types of nucleic acid, are mainly used as site-specific nucleases for the site-specific hydrolysis of a targeted nucleic sequence [35,36]. 

The main advantages of viral vector application are their high transfection efficiency and the vast knowledge of their practical potential, resulting from the countless number of in vivo and in vitro experiments that have been performed with such carriers. In addition, other major features include their ability to productively infect a specific cell, called tropism, provide high expression of the transported mRNA and transfer both dividing and non-dividing cells [37]. However, their high immunogenicity, high production costs and low capacity in terms of genetic material packaging, the temporary expression of the transgene and, most importantly, the risk of insertional mutagenesis, are the main hurdles which limit their use on a larger scale [1,37]. One of the consequences of these disadvantages is a decrease in retroviral vector use in clinical trials (from 28% in 2004 to 19.7% in 2007). This has been caused by the noticeable incidence of severe combined immunodeficiency (SCID) during clinical trials [38]. 

Dendrimers are a promising carrier due to their nano-sized structure and high structural homogeneity [39], and, like proteins, macromolecules, liposomes, micelles and other soluble particles between 10 and 100 nm in size, they may be subject to the EPR, i.e., “Enhanced permeability and retention (EPR) effect” present in the tumor microenvironment [40,41]. However, research on a given effect shows inconsistent results [42]. Over the last decade, hope has increased, due to the existence of the EPR effect. In accordance with this theory, hyperpermeable tumor vasculature allows for the enhanced permeability of large particles, contributing to the "passive targeting" of neoplastic tissue by a specific nanoparticle, not only making it easier for the drug to reach the cancer site, but also allowing for imaging of its microenvironment. However, the existence of the given effect could not be proven in clinical trials, due to the substantial heterogeneity in the tumor environment giving inconsistent results compared to murine systems, where most of the research was conducted [42,43,44,45,46]. The cytotoxicity of dendrimers is another element worth discussion, due to their potential use in future therapies. Generally, it is assumed that the middle generations of dendrimers (3-5G) are less toxic than the higher ones and equally effectively attach genetic material to their surface [37,47]. The other elements, such as the elasticity of dendrimers’ branches [47], internal hydrophobicity [48], surface and core modifications [49,50], have an impact on the efficiency of transfection and, most importantly, the cytotoxicity of dendrimers. Adequate modification of the above-mentioned parameters allows for the maximization transfection efficiency and the simultaneous minimization of cytotoxicity. Another feature offered by dendrimers is the broad range of potential surface modifications that increase their specificity to a selected cellular location. It is assumed that, in the context of potential targeted therapies, these modifications are more clinically relevant than the EPR effect itself [44]. 

According to the latest scientific literature on dendrimers and other nanopolymers, Lipofectamine is the gold standard used to compare transfection efficiency between non-viral vectors [49,51]. It is important to consider the absence of studies directly comparing viral and non-viral vectors in terms of transfection efficiency and abundance of references to both carrier types. It is generally assumed that non-viral vectors have a lower transfection efficiency than viral ones; however, there is no research proving this directly. For this reason, there is a need for studies that will directly compare these two carrier types, in a manner consistent with current methodologies. 

### 1.3. PAMAM Dendrimers

Dendrimers are highly branched, three-dimensional molecules, 1–10 nm in size, with unique physiochemical properties, such as: (1) very low cytotoxicity, (2) capacity to penetrate cells with high efficiency, and (3) an “autofluorescence ability” that allows the investigation of their fate in the cell and the living organism [52,53,54]. The main methods of dendrimer synthesis include the divergent growth method, convergent method, hypercore and branched method, and double and mixed exponential growth method [55], but the most commonly used methods are divergent and convergent [56]. In the divergent method, the synthesis of dendrimer starts at the core, to which individual monomers are added under strictly controlled physicochemical conditions, until the desired dendrimer generation is achieved. With every next generation of dendrimer, the number of branches is doubled. The main advantage of this method is the possibility of achieving a higher reaction efficiency; however, this occurs at the expense of the purity of the synthesized compound and there may be structural defects resulting from the specific nature of the reaction itself [39,57]. In the convergent method, the synthesis starts with the external functional groups of the dendrimer and is executed until all of the groups converge into one point, which is the core of the dendrimer. Using this method, it is feasible to create branches of different sizes in each subsequent synthesized dendrimer generation [58]. Compared to the divergent approach, this provides greater control during synthesis and requires relatively fewer coupling reactions, which guarantees the greater purity of the obtained compound; however, this comes at the expense of reaction yield. Another advantage of this approach is the ability to create asymmetrical dendrimers containing non-uniform functional groups [57,59,60]. Both methods are most commonly used in the synthesis of Poly(amidoamine) (PAMAM) and Poly(propylene imine) (PPI) dendrimers. 

Dendrimers have several other unique properties, which makes them excellent candidate carriers of drugs and genetic material. Due to the controlled process of chemical synthesis, they are monodispersed, which means that they have a uniform shape and size over their entire surface. [37]. Their surface can also be modified by the addition of different kinds of functional groups, which significantly increases their practical capacities by enhancing their solubility and reactivity [61], and creates the possibility of attaching drugs, genetic material, antibodies and signaling molecules, allowing the precise delivery of dendrimer molecules inside the cell [55]. In the context of genetic material delivery, the process of complexing a dendrimer with genetic material reduces the cytotoxicity of the dendrimer itself, which is one of the essential prerequisites for the use of dendriplexes in gene therapies [62]. Moreover, due to their structural and physicochemical properties, they are able to form various structures, i.e., bubbles, microbes, dendrimersomes, discs, cubosomes, helical ribbons and various forms of aggregates. This creates a unique opportunity to use dendrimers as carriers for many types of drug, and for controlled drug release to target cells, depending on a specific external stimulus [63,64]. Another unique property of dendrimers is the chemical and physical stability resulting from their chemical structure [52]. Furthermore, the stability of the dendrimer–substrate bond can be modified by the alteration of the pH solution and concentration of the dendrimer itself. This approach allows for the delivery of drugs and genetic material inside the cell, providing for their accumulation at the appropriate target location, where, under standard conditions, this would be difficult to achieve [56,65,66,67]. Th efficacy and effectiveness of genetic material delivery of PAMAM dendrimers have been well characterized in vitro and in vivo studies [1,30,68,69]. They have demonstrated that carriers such as PAMAM dendrimers are highly effective at delivering genetic material (siRNA, miRNA plasmids) into tumor cells, causing a specific therapeutic effect in in vitro and in vivo systems.

**Figure 1:**
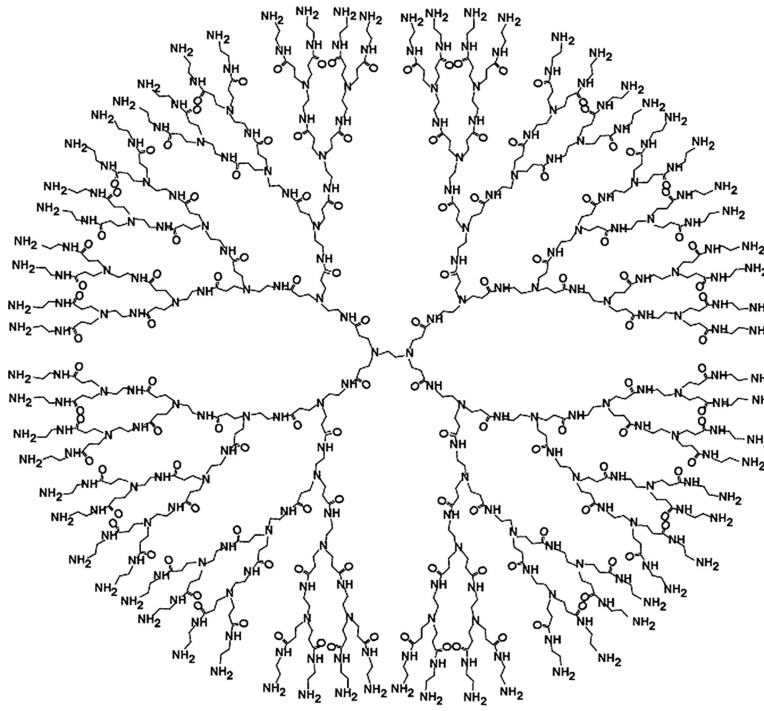
Chemical structure of poly(amidoamine) (PAMAM) G4. With the permission of [77].

Polyamidoamine (PAMAM) dendrimers are one of the most frequently used nano-sized molecules in science, produced on an industrial scale (Figure 1). In 1985, A. Tomalia published an article in *Nature* reporting on their synthesis [70]. PAMAM was one of the first dendrimer families to be fully characterized, synthesized and commercialized. Due to the numerous possibilities of modification of its core and surface groups and its highly symmetrical structure, hydrophilic properties, high biocompatibility and non-immunogenic properties, PAMAM dendrimers are extensively used in research on targeted drugs and genetic material delivery [71,72]. From a structural perspective, PAMAM dendrimers comprise a core, internal branches, an empty internal space between branches and surface groups at their ends. During the synthesis of dendrimers, ethylenediamine, diaminododecane, diaminoexane, and diaminobutane are used as hydrophobic cores [73,74,75]. The most commonly used compounds creating internal branches are methyl acrylate and ethylenediamine, while the surface groups are most often built of compounds rich in amide or carboxylic groups [76]. 

Considering the extensive knowledge of intracellular processes involved in dendrimer delivery to the cell, and all the modifications facilitating this process, it seems surprising that it has not yet been possible to extrapolate the results from in vitro and in vivo studies to clinical settings. Despite intensive scientific efforts, an effective gene therapy using dendrimers as the main carriers of genetic material has still not been developed. However, the promising results of research on PAMAM, PPI and Poly-l-lysine (PLL) dendrimers indicate that this trend may change soon. This review examines one of the most thoroughly studied dendrimers, PAMAM, and critically summarizes the current knowledge from the research into finding an effective and efficient genetic material delivery system in cancer gene therapy.

## 2. Recent Advancements in Cancer Gene Therapy Studies Using PAMAM Dendrimers

**Figure 2:**
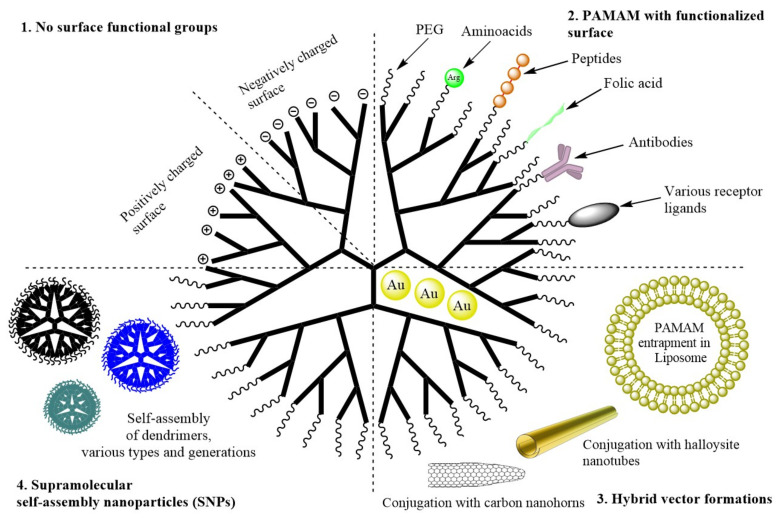
Schematic representation of the PAMAM dendrimer modification strategies covered in the review.

During the last 10 years of research on PAMAM dendrimers, four research strategies have been distinguished (Figure 2):

(1) No surface functional groups;
(2) PAMAM with functionalized surface (PEG, amino acids and peptides, antibodies, hydrophobic particles, folic acid and other polymers);
(3) Hybrid vector formations (conjugation into non-dendritic nanomolecular polymers, entrapment of PAMAM in liposome, entrapment of gold nanoparticles);
(4) Supramolecular self-assembly nanoparticles (SNPs).

### 2.1. No Surface-Functional Groups

This group includes quite a large portion of the of preliminary research on PAMAM dendrimers, which was particularly noticeable prior to 2010, since the main focus of the research was aimed at the characterization of dendrimers, analysis of zeta potential, hydrodynamic diameter and application of basic biomolecular assays, such as cytotoxicity tests and the ability to transport genetic material to different cell lines. In the following years, the more frequent use of PAMAM dendrimers with at least one type of functional surface modification has become noticeable, with a similar methodological approach adopted in "primary" studies, which are additionally enhanced by the use of various types of chemotherapeutics and anticancer drugs and interference RNAs, aimed directly at different molecular targets, whose cumulative effect began to be studied not only in vitro, but also in vivo.

**Figure 3:**
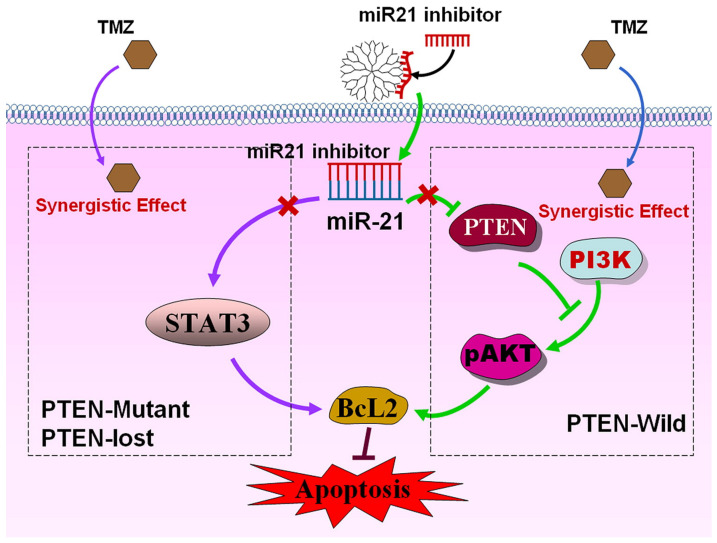
Mechanism of combination therapy for miR-21i and TMZ. miR-21i was delivered by PAMAM into the cells and coordinated with miR-21, leading to inactivation of miR-21. For LN229 cells (PTEN-wild), the inactivation of miR-21, which is inhibited by the simultaneous miR-21i and TMZ treatment, targets and reduces the expression of PTEN, blocks the PI3K-pAKT pathway, induces an increase in apoptosis, and leads to synergistic antiproliferative activity. For U251 (PTEN mutant) and U87 (PTEN-lost) cell lines with high STAT3 expression, the synergistic effect was achieved by sequence treatment of the two drugs. Reprinted with permission from [78], copyright (2021) American Chemical Society.

In one representative study, Temozolomide (TMZ) and PAMAM G5 dendrimers transporting microRNA 21 inhibitor (miR-21i) were used to test the anticancer efficacy in the treatment of glioma on three cancer lines, LN229 (Phosphatase And Tensin Homolog wild variant; PTEN-wild), U87 (PTEN-lost), and U251 (PTEN mutant). Since differences in phenotypic context may affect the consistency of results, three cell lines were used instead of one, with different phenotypic features. The research hypothesis was to determine the relationship between the sequential-dependent co-delivery of miRNA and TMZ and the effectiveness of therapeutic intervention measured by the percentage of apoptotic cells and changes in PTEN expression and PTEN-dependent pathways on three lines of different phenotypes. The study demonstrated that the best anticancer effect of mi-R21i and TMZ administration was observed for the LN229 cell line (PTEN mutant), whilst mi-R21i−/+ 4 h after TMZ co-delivery was most effective in U251 (PTEN mutant) and U87 (PTEN lost) cell lines. This effect is particularly noticeable in the case of loss of PTEN function and maximum inhibition of Signal Transducer And Activator Of Transcription 3 (STAT3) expression and level of phosphorylated STAT3, which leads to the additional reinforcement of TMZ and miR-21i action, induction of apoptosis and strong inhibition of proliferation (Figure 3) [78].

In another study targeting the same molecular target, PAMAM G5 was used to co-deliver antisense-miR-21 oligonucleotide (as-miR-21) and 5-fluorouracil (5-FU) to a U251 human glioma cell line in order to increase sensitivity to the 5-FU chemotherapeutic agent. 5-FU is a widely used anticancer agent in the treatment of breast cancer and other types of cancer. It is an uracil analogue comprising a fluorine atom in the C-5 hydrogen position. Once it penetrates the cell, it attaches to DNA or RNA, leading to a disturbance in nucleoside metabolism, inevitably causing apoptosis [79]. The study demonstrated that PAMAM G5 dendrimers were able to compact as-miR-21 and 5-FU simultaneously, forming 100 nm complexes. The results of this study indicated a synergistic effect on cancer cells, increasing the percentage of apoptotic cells, significantly reducing proliferation and their migration. as-miR-21 substantially increased the anticancer activity of 5-FU, which demonstrates that the combined as-miR-21 and 5-FU therapy, supported by PAMAM G5, is a viable therapeutic option [80].

In another study, a relatively interesting therapeutic approach has been employed, involving the use of Herpes Simplex Virus Thymidine Kinase (HSV-TK)/ganciclovir (GCV) system (HSV-TK/GCV) fused with the Connexin 43 (Cx43) gene in suicide gene therapy and gemcitabine to enhance the anticancer effect in the treatment of locally advanced or metastatic prostate cancer. The HSV-TK/GCV was fused together with Cx43 to form a recombinant plasmid, in order to enhance the default system’s performance. The objective of the study involved the transfer of recombinant plasmid by means of PAMAM G5 dendrimers, which would allow the safe delivery of the double-enhanced system without triggering an immune response, and enable the preservation of the "bystander effect", which is crucial in gene suicide therapies. It has been demonstrated that the HSV-TK/GCV-Cx43 system effectively suppressed the proliferation of prostate cancer cells, triggered an apoptosis and led to a significant reduction in tumor size in the prostate cancer nude mice model with human prostate cancer cells (PC-3) [81].

**Figure 4:**
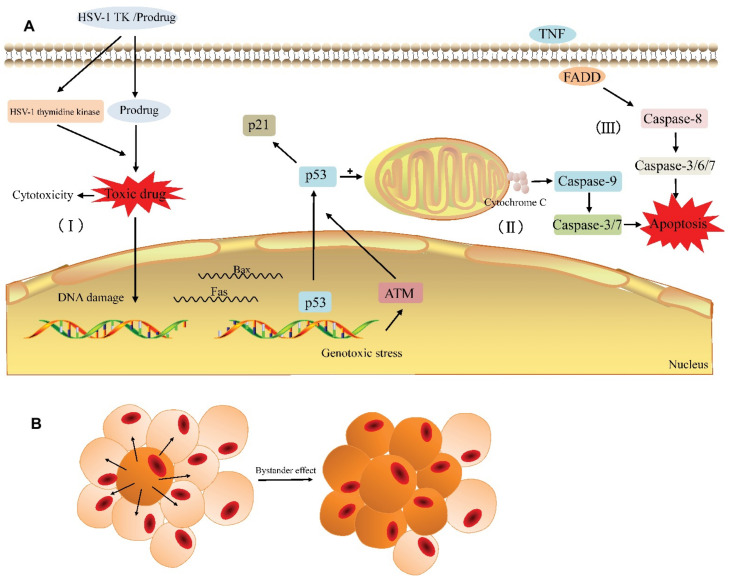
General principle of the HSV-TK suicide gene system. (**A**) The HSV-TK/prodrug-system-induced cytotoxicity and apoptosis in tumor cell. (I) The HSV-1 TK gene coding for thymidine kinase is delivered to the target cell. The expression of HSV-1 thymidine kinase and cellular kinases allows a prodrug (GCV) to be activated as the toxic drug (GCV-TP) in the cell and damage the DNA of the target cell. (II) Gene therapy induces endogenous apoptosis by activating p53 signaling pathway in the target cell. (III) Gene therapy induces exogenous apoptosis. (**B**) The bystander effect induces the death of adjacent cells. With the permission of [85].

Suicide gene therapy is a treatment-oriented strategy using genes that induce apoptosis in cancer cells, while keeping healthy cells untouched. It has been successfully tested in experimental anticancer therapies in vitro and in vivo, such as intestinal, liver, lung, brain and bladder cancer, as well as in clinical trials in glioma therapy [82]. The fundamental principle is based on the introduction of a non-toxic pro-drug, which undergoes transformation into a cytotoxic agent in the cancer environment, initiated by the intracellular expression of a suicide gene of bacterial or viral origin, leading to such a transformation [83]. The HSV-tk/GCV system consists of the thymidine kinase gene of the herpes simplex virus (HSV-tk), which converts ganciclovir (GCV) to ganciclovir monophosphate. In the cancer cell cytoplasm, ganciclovir monophosphate is converted by the enzymatic system to cytotoxic ganciclovir triphosphate (Figure 4). The consequence of HSV-tk/GCV action is a delay in the S and G2-phase, which results in the suppression of proliferation process, activation of cascade-8 and Chk1, causing cell death [84,85]. Cx43 is a gap junction protein composed of 382 amino acids with a molecular weight of 43 kDa [86]. It has been observed that Cx43 is underexpressed in tumor cells, which positively correlates with progression, cancer metastases and worse survival; however, the data are inconsistent for different types of cancer [87,88]. 

The only drawback of these studies is that no reference has been made with respect to the non-cancerous cell line, and the cytotoxicity of the complex itself has not been determined, which is currently a major concern for unmodified PAMAM dendrimers.

### 2.2. PAMAM with Multi-Functionalized Surface

This group includes the vast majority of in vitro and in vivo studies on PAMAM dendrimers, aimed at anticancer gene therapy in breast cancer [89,90,91,92,93,94], hepatocellular carcinoma [95,96,97,98,99], gastric cancer [100,101], cervical carcinoma [102,103], ovarian cancer [104,105], lung cancer [106,107], glioma [69,108,109,110,111,112,113,114,115,116], colon adenocarcinoma [117,118,119,120], thyroid cancer [121] and head and neck squamous cell carcinoma [122,123]. The most frequently used cell lines in research are breast cancer, glioma, colon and hepatocellular carcinoma, whilst the most commonly used dendrimers are those of the 4th and 5th generation. The potential reason for this trend has been described above, and is related to the optimal ratio of the cytotoxicity to transfection efficiency. 

The attachment of polyethylene glycol (PEG) to the nanocarrier surface is one of the most widely used surface modifications in nanomedicine. Moreover, PEG is capable of enhancing the pharmacokinetics of the conjugate by increasing its circulation half-life by reducing interactions with serum proteins [98,124], and increases the likelihood of nanocarrier penetration into tumor tissues by facilitating passive targeting via the EPR effect [125]. Pegylation also reduces the immunological response and increases the solubility of the conjugated molecule [126]. PEG coating makes the nanocarrier system hydrophilic, allowing water molecules to form hydrogen bonds with oxygen molecules on the PEG, which creates a hydrated film around the nanocarrier, thereby facilitating a mitigation of the immune response to the nanocarrier [127,128]. 

Regarding PAMAM dendrimers, despite the obvious advantages of pegylation, such as the mitigation of negative properties of "naked PAMAM", a reduction in the internalization efficiency by target tissues, e.g., endothelium, hepatocytes, also occurs [129]. In order to address this issue, scientists have been decided to conjugate additional molecules to its surface, such as amino acids, peptides, cyclic RGD, lactobionic acid. 

In one of these studies, angiopep-2, a ligand of lipoprotein receptor-related protein 1 (LRP1) present in glial cells, was conjugated with the surface of a pegylated PAMAM dendrimer (PAMAM-PEG-Angiopep) in order to target glioma cells and deliver tumor-necrosis-factor-related apoptosis-inducing ligand (TRAIL) to the C6 glioma cell line and brain-tumor-bearing ICR and nude mice. TRAIL is a cytokine that induces apoptosis in tumor cells via the p53-independent pathway by binding to death receptors 4 (TRAIL-R1) and 5 (TRAIL-R2/KILLER). The glioma cells show overexpression of the TRAIL receptor. PAMAM-PEG-Angiopep, complexed with TRAIL plasmid, displayed excellent blood–brain-barrier penetration ability and a favorable biodistribution and pharmacodynamic profile in vivo, thus emerging as a promising nanocarrier for targeted therapies in glioma [112]. 

In another study, cyclic Arg–Gly–Asp–d-Phe–Cys (RGDfC) peptides were used as an additional surface modification, in addition to PEG. PAMAM-ABP dendrimers were conjugated with cyclic RGDfC and used to deliver shVEGF-containing plasmid to breast cancer cells (MCF7) and pancreatic cancer cells (PANC-1), where a significant decrease in survival after the application of a given vector was observed [89]. RGD is a short peptide motif, consisting of three amino acids, such as arginine, glycine and aspartic acid (Arg–Gly–Asp). It has been successfully applied as a surface modification in many polymer, lipid and peptide carriers [130]. Linear and cyclic peptides, such as RGD4C and RGDf4, have been shown to possess high affinity to α_v_β_3/5_ integrins. After the activation of the Vascular Endothelial Growth Factor (VEGF) receptor, they undergo cross-activation, which leads to an angiogenesis process. This provides an excellent anchor point for targeted anticancer therapies, where the phenomenon of angiogenesis plays a critical role in neoplastic proliferation. Due to the binding of RGD peptides to integrins, it is possible to introduce siRNA against the VEGF receptor, which will lead to a reduction in its expression and the consequent inhibition of angiogenesis and cancer death [131].

In another study, PAMAM dendrimers conjugated with lactobionic acid (GAL) and PEG were used in order to deliver siRNA against astrocyte elevated gene-1 (AEG-1) to human hepatocellular carcinoma xenograft mice. The addition of GAL increased the specificity of dendrimers against hepatocellular carcinoma cells, by using asialoglycoprotein as a molecular target. This is a receptor which is overexpressed in liver cancer cells; hence, it represents a good candidate for a molecular target in hepatocellular carcinoma-targeting therapies [98]. 

In targeted brain tumor therapy, the transferrin (Tf) receptor is a potent molecular target due to its overexpression in the blood–brain barrier cells, which has been demonstrated in the experiment involving PAMAM dendrimers conjugated with Tf via PEG. In the study, PAMAM-PEG-Tf dendrimers were combined with the TRAIL-encoding plasmid. PAMAM-PEG-Tf dendrimers, along with TRAIL-encoding plasmid, have shown increased cellular uptake, exclusive expression of plasmid in the C6 cancer cell line and increased survival of mice with glioma (28.5 days) as compared to control (25.5 days) [115].

Conjugation with antibodies is another example of surface modification increasing cellular specificity. In one study, dendrimers conjugated with epidermal growth factor (EGF), human serum albumin (HSA) and nimotuzumab antibody (h-R3) were used to deliver siRNA against polo-like kinase-1 (PLK1) to the EGFR-overexpressing hepatocellular carcinoma cell line (HepG2) and tumor-bearing BALB/c nude mice. PAMAM dendrimers conjugated with EGF, HSA and h-R3 ligands exhibited increased particle size, decreased zeta potential, lower cytotoxicity and increased target-gene-silencing performance relative to HSA-dendriplex, h-R3 dendriplex, EGF-dendriplex and "naked" dendrimers [97]. H-R3 is a humanized monoclonal antibody against the epidermal growth factor receptor (EGFR). It displays pro-apoptotic, anti-proliferative and anti-angiogenetic properties [132]. The addition of EGF to the dendrimer surface significantly increases cellular specificity, successfully promoting enhanced gene expression silencing in cells with a high expression of the EGF receptor [133], whereas the addition of HSA facilitates the internalization of dendrimer via endocytosis. This is associated with the presence of the endothelial protein Gp60, located in the caveolae. Gp60 is a membrane protein which binds albumin and facilitates its uptake through endothelium and epithelial barriers [134]. 

A further, frequently used modification is folic acid, also known as vitamin B9. As a ligand, it is non-immunogenic, characterized by its functional stability, defined manufacturing process and excellent permeability to cells via internalization pathways. The receptor for folic acid is present in an elevated amount in cancer cells [135]. It has been proven that the expression of a given receptor reaches up to 100–300 times higher than in healthy tissues [108,136], and this has found practical application in the design of a dendrimer conjugated with folic acid. One study showed that PAMAM G4 dendrimers conjugated with folic acid show selective and increased uptake in head and neck squamous cell carcinomas (HN12)-overexpressing folic acid receptor. Moreover, in combination with siRNA against VEGFA, they caused a reduction in tumor growth after a single dose in mice [123]. 

### 2.3. Hybrid Vector Formations

This group includes more complex nano-systems, in which other nanopolymers or gold particles are added, in addition to the conjugation of various surface ligands with dendrimers. Some of the most interesting hybrid nano-systems are those entrapping gold nanoparticles and conjugating surface ligands. The entrapment of gold particles inside dendrimers is used on the basis of the assumption that gold tends to strengthen the inner structure of the dendrimer, allowing for the preservation of the 3D spatial structure, which is essential for the successful compaction of genetic material on the surface of the dendrimer, without raising concerns that, under the influence of the cellular environment, the dendrimer will lose its spatial structure [137]. 

In one study, dendrimers entrapping gold nanoparticles (Au DENPs) were conjugated with PEG for the simultaneous loading and delivery of miR-21i and gemcitabine (Gem). It has been shown that a given nano-system permits the encapsulation of 30 gemcitabine molecules per dendrimer and binds genetic material at a low N/P ratio. The co-delivery of Gem and miR-21i via Au DENPs increases in vitro therapeutic efficiency, apoptosis of pancreatic cancer cells (SW1990) and increased efficacy of cancer inhibition in vivo, in comparison with the separate administration of Gem [138]. Gemcitabine (Gem; 2’, 2’-diflurodeoxycytidine) is a nucleoside analogue that incorporates itself into the DNA, causing irreparable genetic damage, leading to the inhibition of DNA synthesis and cell apoptosis. This is applied to patients with locally unresectable advanced or metastatic pancreatic cancer, whereas miR-21 is one of the most expressed miRNAs in a given type of cancer. It modulates, among others, the apoptosis of cancer cells, making them less susceptible to Gem treatment. A potential reason for this is the activation of the PI3K/Akt pathway through a reduction in phosphatase and tensin homologue (PTEN) protein expression, which, in turn, leads to the lowering of pro-apoptotic B-cell lymphoma-extra-large (Bcl-xL) protein expression. The simultaneous application of miR-21 and Gem inhibitor in combination with Au DENP gives hope for more effective pancreatic cancer treatment [138].

**Figure 5:**
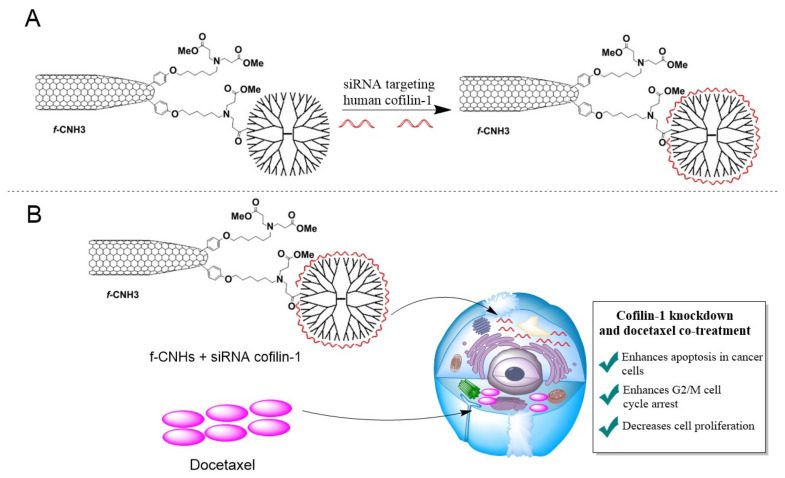
Schematic representation of an experiment involving the use of f-CNH3 hybrid nanovector. (**A**) Conjugation of f-CNH3 with siRNA targeted against human cofilin-1, a key protein in the regulation of cellular cytoskeleton. (**B**) Synergistic effects of siRNA against cofilin-1 and docetaxel on the LNCaP prostate cancer cell line. The f-CNH3-transported siRNA is released in the cell and inhibits cofilin-1 protein expression, leading to the "sensitization" of tumor cells to the effects of docetaxel treatment, resulting in widespread apoptosis, cell-cycle arrest at the G2/M phase, and subsequent inhibition of tumor cell proliferation. Adapted with the permission of [139].

In one study, an interesting nano-system was developed, in which PAMAM and carbon nanohorns (CNHs) were conjugated (f-CNH3) to deliver siRNA against cofilin-1, the protein responsible for regulating cellular cytoskeleton formation, in order to enhance the anti-tumoral properties of docetaxel in the LNCaP prostate cancer cell line (Figure 5). Docetaxel is an anti-cancer drug used to treat prostate cancer. The characteristic feature of this nano-system is its ability to release bounded siRNA when there is an excess of heparin. In addition, it effectively prevents the degradation of siRNAs by RNAs, which is a prerequisite for effective targeted and combined anticancer therapies. After the application of a given nano-system with the drug and siRNA, a 20% decrease in the expression of mRNA cofilin-1 was observed compared to the controls, while, with docetaxel, a synergistic effect of the increased concentration-dependent activation of caspase-3, cell death and cell cycle arrest was observed, as compared to the use of docetaxel alone [139].

**Figure 6:**
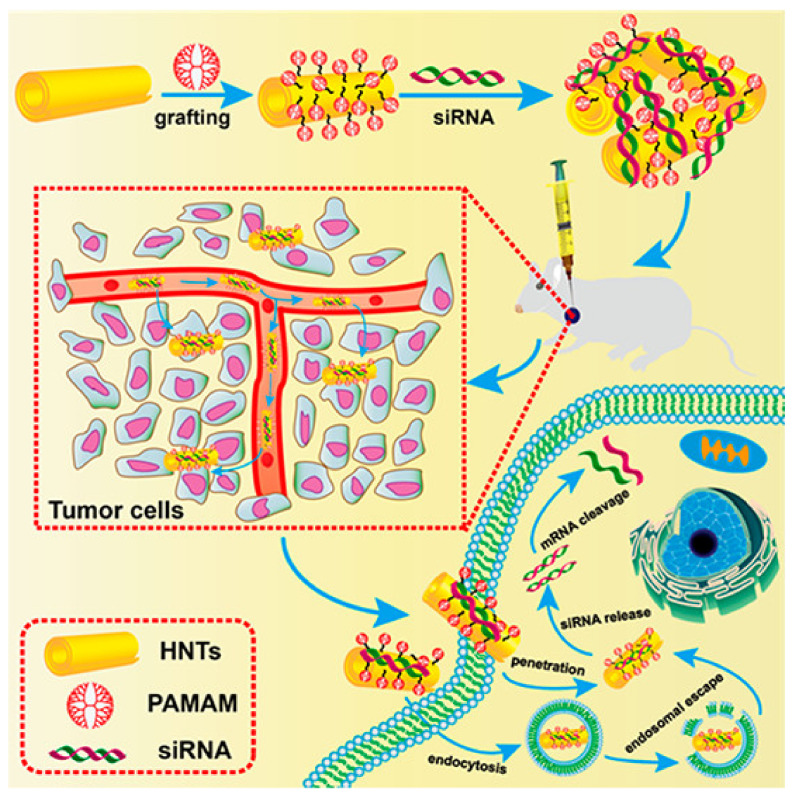
Schematic illustration for the preparation of PAMAM-g-HNTs/siRNA complex and its intracellular process in cancer cells. Reprinted with permission from [68], copyright (2021) American Chemical Society.

Another equally interesting hybrid vector is polyamidoamine-grafted halloysite nanotubes (PAMAM-g-HNTs). This is a nano-system consisting of halloysite nanotubes coated with PAMAM G4 dendrimers (Figure 6). Halloysite nanotubes (HNTs) are naturally occurring cylindrical clay nanomaterials, composed of multi-rolled aluminosilicate kaolin sheets. They exhibit beneficial properties as carriers of genetic material and drugs, such as a high dispersion stability, good biocompatibility, high reactivity, and the presence of multiple hydrophilic hydroxyl groups for functionalization, and proper nanoscale dimensions (~50 nm outer diameter) [140]. In the aforementioned study, PAMAM-g-HNTs were used to deliver siRNA against a vascular endothelial growth factor (VEGF) to breast cancer cell lines (MCF-7 and 4T1) and a 4T1-bearing mice model. It has been demonstrated that PAMAM-g-HNTs present good cytocompatibility and high cellular uptake, and effectively suppress mRNA VEGF expression in vitro and inhibit tumor growth in vivo, in comparison with Lipofectamine [68].

In another promising study, PAMAM G4, conjugated with PEG, 10-bromodecanoic acid (10C) and non-covalently attached to the dendrimer surface anti-nucleolin aptamer (AS1411), was used in order to deliver shRNA against Bcl-xL in a lung-cancer cell line (A549). In this study, a significant improvement in the transfection efficiency and selectivity toward lung-cancer cells was observed after application of the modified PAMAM-10C-PEG vector linked to the AS1411 aptamer, compared to a vector without the aptamer. In addition, the significant suppression of Bcl-xL expression after shRNA application was observed compared to the control containing shRNA-Bcl-xL enclosed in the aptamer-free nanocarrier [141]. The incorporation of an alkyl agent such as 10-bromodecanoic acid increases the solubility of the dendrimer, and additional conjugation with PEG further increases its capacity for efficient transfection. AS1411 is a 26-base guanine-rich DNA aptamer, which is responsible for the binding of nucleoin, a phosphoprotein that is highly expressed in many cancers, including acute myeloid leukemia, breast cancer and lung cancer. AS1411 is internalized after binding to the cell surface via the nucleolin receptor and may inhibit nucleolin binding to mRNA-encoding anti-apoptotic B-cell lymphoma 2 (BCL2), thereby leading to mRNA destabilization and nucleolytic degradation, which, in turn, may result in a reduction in BCL2 expression at protein level, and the induction of apoptosis. However, this effect is presumed and, more research is needed to confirm it, while one study found that cell death caused by a given aptamer occurs via methuosis, a non-apoptotic pathway of cell death, which is also a poorly understood issue [142,143,144]. However, the effectiveness of aptamer as a targeting ligand is undeniable, which has been proven in research [144].

In another study, a hybrid nano-system of PAMAM and phospholipids surrounding a single dendrimer molecule, delivering siRNA against the multi-drug resistance 1 receptor (MDR1), was developed. This system was designed in order to reverse multi-drug resistance, whereas the very aim of the study was to create a system that would eliminate the primary disadvantages of PAMAM dendrimers, such as the instability of encapsulation, low transfection efficiency and inefficient cellular internalization. The advantages of the liposomal system include its potential for drugs and DNA binding and biofilm affinity. Negatively charged phospholipid groups easily adhere to positively charged PAMAM surface groups, surrounding the entire dendritic molecule. The lipid molecules associated with the dendrimer surface easily interact with the cell membrane, facilitating the internalization of the entire nanocomplex via the endocytotic pathway, resulting in an increased transfection efficiency, and thus enhancing gene silencing. In this study, after administration of a given complex combined with siRNA against MDR1 in the breast cancer cell lines (MCF-7 and MCF-7/ADR), an increased cell apoptosis, impact on cell-cycle regulation, decreased p-gp expression and increased sensitivity to doxorubicin (DOX) and paclitaxel (PTX) were observed, which manifested in a higher intracellular accumulation of DOX and PTX, and the inhibition of tumor cell migration, as compared to PAMAM-siRNA without a phospholipid envelope [145]. 

Tariq et al. also described the development of PAMAM lipodendriplexes, and comprehensively studied their properties in vitro (2D and 3D cell culture) and in vivo. Lipodendriplexes showed reduced cytotoxicity, and improved gene transfection in vitro and in vivo. Shielding of the terminal dendriplex amino groups increased the biocompatibility and decreased the cytotoxicity of the complex in the in vivo environment. Moreover, lipodendriplexes showed an improved transfection efficiency and safety profile compared to naked dendriplexes, both in vitro and in vivo. However, further studies on various in vivo models using functionalization of the lipodendriplex surface with receptor-specific ligands are required for the development of a full pharmacokinetic and pharmacodynamic model for further use, e.g., in cancer gene therapies [146].

### 2.4. Supramolecular Self-Assembly Nanoparticles (SNPs)

Supramolecular self-assembly nanoparticles are more complex than hybrid nano-systems. Their distinctive feature is their ability to self-assemble into larger, organized structures via hydrophobic interactions, hydrogen bonding, and metal–ligand interactions [147,148,149]. Their main advantage is their almost unlimited number of possible combinations, which possess the positive features of the individual elements in the system, while eliminating the negative ones. They have the potential to be deployed in a wide range of possible applications in many areas, such as the production of nanomaterials, diagnostics, imaging, biosensors, biomedical applications and drug/gene delivery. Despite their promising properties and high level of practical potential, several challenges need to be addressed in the large-scale implementation of these nano-systems, including improvements in protocols to obtain particles of a similar size, their stability in an aqueous environment, the determination of drug/genetic material-loading capacity ratios, and their immunogenicity and biocompatibility. PAMAM dendrimers and other types of dendritic nanopolymer have already been used to create supramolecular self-assemblies [150,151].

**Figure 7:**
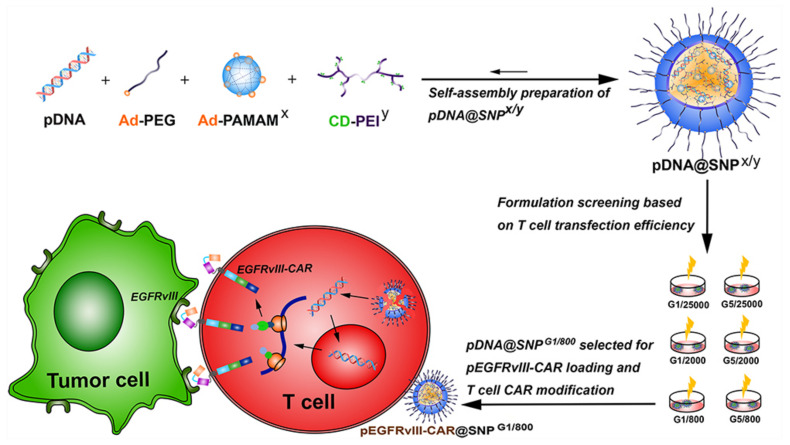
Schematic illustration of the preparation and formulation screening of pDNA@SNPs for pEGFRvIII-CAR loading and T cell CAR modification. Abbreviations: pDNA, plasmid DNA; Ad-PEG, adamantane-grafted poly(ethylene glycol); Ad-PAMAMx, adamantane-grafted polyamidoamine dendrimer (x: PAMAM dendrimer generation); CD-PEIy, cyclodextrin-grafted branched polyethylenimine (y: PEI molecular weight); SNPs, self-assembled nanoparticles; EGFRvIII, epidermal growth factor receptor variant III; CAR, chimeric antigen receptor; pEGFRvIII-CAR, epidermal growth factor receptor variant III-chimeric antigen receptor expression plasmid. With the permission of [153], copyright (2021) Dove Medical Press.

A rather interesting study using the phenomenon of supramolecular self-assembly was performed using adamantane-grafted polyamidoamine (Ad-PAMAM), cyclodextrin-grafted branched polyethyleneimine (CD-PEI), and adamantane-grafted (Ad-PEG). One of the major advantages of CD-PEI and AD-PAMAM application in the formation of SNP is their positive surface charge, which allows for effective drug and genetic material encapsulation due to the charge–charge interaction and their release during endocytosis, by means of the proton-sponge effect [152]. The aim of this study was to create Chimeric Antigen Receptor T cells (CAR-T) with EGFRvIII-Chimeric Antigen Receptor (EGFRvIII-CAR), and to test their efficacy on a cell line overexpressing the EGFRvIII receptor. In the first step, the least toxic and most efficient self-assembly combination of three dendrimers was chosen from a set of several different molecular masses. In the second step, the set most beneficial to the expression of EGFRvIII-CAR in Jurkat T cell line, by means of cell line transfection with plasmid containing the appropriate gene, was chosen. In the third stage, previously created EGFRvIII-CAR-T cells were used to recognize human hepatocarcinoma HuH7 cells with EGFRvIII overexpression. It has been shown that the most effective and least toxic supramolecular self-assembly complex was the one composed of PAMAM G1 and PEI, with a molecular weight of 800 Daltons. The complex successfully delivered the plasmid with EGFRvIII-CAR to Jurkat T cells, which specifically recognized EGFRvIII-positive cancer cells (Figure 7). This study has demonstrated the high transient CAR transfection efficiency and low toxicity towards Jurkat T cells, which, in the view of the research team, can be used in future in vivo and ex vivo studies of the given complex, forming a basis for potential clinical trials [153].

**Figure 8:**
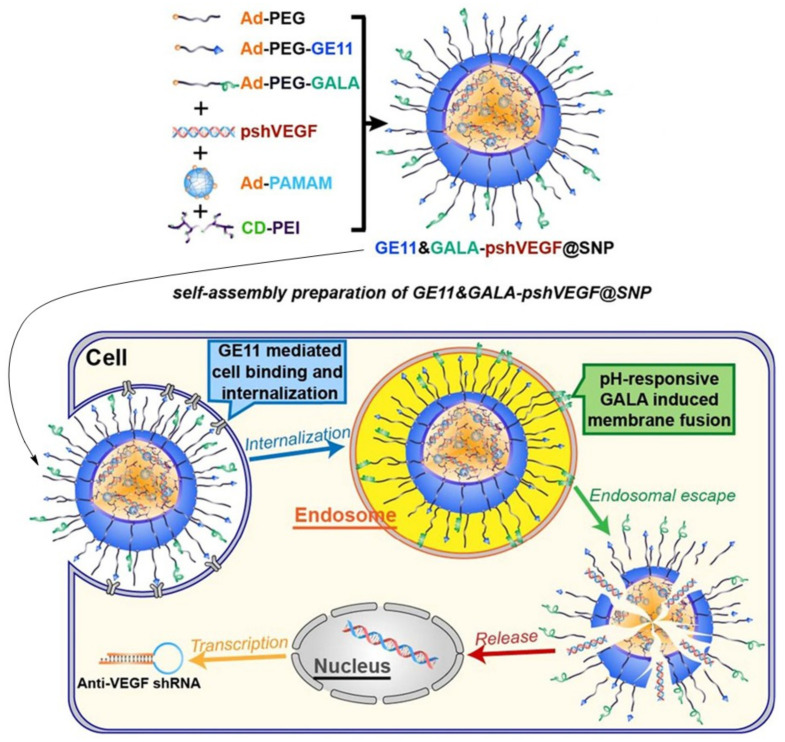
Schematic representation of the preparation and formation of self-assembly nanoparticle and transfection processes inside the cell. With the permission of [152].

In another study using the self-assembly phenomenon, roughly similar elements were used to the previously described study, i.e., adamantane-grafted polyamidoamine (Ad-PAMAM), cyclodextrin-grafted branched polyethyleneimine (CD-PEI) and adamantane-grafted (Ad-PEG). In order to increase the tumor specificity of the SNP complex toward lung cancer cells, the SNP complex was conjugated with an EGFR-targeted peptide GE11 and pH-sensitive fusogenic peptide GALA, in order to form a functional complex for genetic material delivery (GE11&GALA-DNA@SNPs). The GE11&GALA-DNA@SNPs complex was used to deliver small a hairpin RNA expression cassette against vascular endothelial growth factor (VEGF) to the A549 cell line and tumor-bearing mice. This complex successfully delivered shRNA against VEGF, reducing its expression compared to Lipofectamine 2000, whereby, in a lung cancer mouse model, the growth and size of the tumor was significantly reduced after shVEGF was delivered with GE11&GALA-pshVEGF@SNPs (Figure 8). This also showed no significant in vivo cytotoxicity. However, as indicated by the authors themselves, no significant difference was observed regarding in vivo tumor growth inhibition after GE11-pshVEGF@SNP and GE11&GALA-pshVEGF@SNPs, which is the starting point for further studies [152]. The GE11 is a dodecapeptide EGFR-specific ligand that binds to the receptor without activating it [154]. GALA is a synthetic peptide with a repetitive "EXLA" motif, which changes its structure as a function of the pH of the solution from a random coil (pH < 5) to an amphipathic alpha-helix (pH > 5) [155]. GALA binds to lipid membranes, and at a sufficiently high concentration on the surface of the SNP complex, it forms interstitial pores. The internalization of the SNP complex via endocytosis, and the low pH inside the endocytic bubble, activate GALA and may promote endocytic escape [156]. 

In another report on the development of SNP carriers, core-shell tecto dendrimers (CSTDs) were designed for the delivery of the microRNA 21 inhibitor (miR-21i) and doxorubicin to the breast-cancer cell line (MDA-MB-231). CSTDs are composed of cyclodextrin-grafted G5 PAMAM dendrimers, which constitute the core of the complex, and adamantane (Ad)-grafted G3 PAMAM dendrimers as shell elements. The aim of the study was to investigate CSTD complexes for their applicability in combined miR-21i and doxorubicin therapy. The CSTDs exhibit large internal spaces and excellent gene compacting properties, allowing them to bind miR-21i by means of electrostatic interactions and doxorubicin via entrapment in the internal spaces of PAMAM G3, constituting the shell layer. CSTDs transport miR-21i and doxorubicin at a low N/P ratio of 10, promoting effective delivery to MDA-MB-231 cells, and leading to the suppression of miR-21 expression and positive regulation in proapoptotic and migration-regulating genes, resulting in proliferation and migration inhibition, while the co-delivery of doxorubicin significantly increases this effect compared to "naked" doxorubicin [157]. Despite promising results, further studies are required in order to assess the efficacy of the CSTD complex in vivo and ex vivo, in combined anticancer therapies.

Table 1 provides a brief summary of the above-mentioned studies in terms of the dendrimer-based nanosystems’ classification, with regard to the type of modification and the nature of the delivered genetic material.

## 3. Recent Advancements in Cancer Gene Therapy Studies Using PAMAM Dendrimers: Author’s Insights

The history of gene therapy dates back to the 1970s, when an article titled "Gene therapy for human genetic disease?" was published in *Science* [161]. The very idea of the introduction of external DNA into the patient’s body to cure rare genetic defects constituted the beginning of gene therapy. Over the last 50 years, gene therapy has evolved with the development of many research techniques and breakthroughs in the field of molecular biology, genetics and immunology, extending its focus to not only rare diseases, but, above all, cancer and broadly defined metabolic, immunological and civilization disorders, such as diabetes, obesity, atherosclerosis, cardiovascular and autoimmune diseases, and the most prevalent neurodegenerative disorders such as Parkinson’s and Alzheimer’s disease [162,163]. As the years progressed, new breakthroughs in the field of molecular genetics were made, such as the existence of interference RNAs, different types of non-coding RNAs, molecules that are part of the transcriptome and have associations with the proteome. With the recently discovered epigenome, attempts were made to use these newly found molecules for therapeutic purposes. One of the biggest concerns that has overshadowed the extensive use of gene therapy to date is the side effects of viral vectors, which pose a real risk of mutations and serious immunological reactions that create a risk to patient’s health and life. The best possible alternative, adeno-associated viruses, are currently being used, which eliminate the risk of random insertions and unwanted mutations, but carry a further risk of immunological reactions in the host body.

The conceptual approach, involving the transfer of newly discovered types of genetic material by means of non-viral carriers such as dendrimers, was born about two decades ago. The first "genetic" experiments with PAMAM dendrimers date back to 1993, when they were shown to be effective at delivering luciferase and beta-galactosidase-containing plasmids into mammalian cell lines [164]. In the following years, the field of action gradually expanded through investigations with PAMAM on normal and neoplastic cell lines, and the transferal of mRNA, plasmids, siRNA, ASO and other types of genetic material into the cells. In the last 10 years, PAMAM dendrimers have received considerable research interest, resulting in experiments demonstrating their usefulness as carriers of therapeutic genetic material in in vitro and, above all, in vivo configurations. This is particularly important because there is a strong demand for efficient and safe carriers that are able to transport not only the genetic material itself, but also drugs.

The last 10 years of research on PAMAM dendrimers have revealed quite interesting experimental arrangements, where surface modifications and conjugation with other large polymers became increasingly common. It was decided to transport not only the genetic material itself, but also cytostatic drugs. The most frequently used genetic material was "naked" and plasmid siRNAs, miRNAs, as well as short hairpin RNAs (shRNAs) directed towards a specific molecular target, and plasmids providing suppressive proteins (p53) or apoptosis inductors (TRAIL, apoptin). A method frequently adopted in gene therapies, regardless of the type of gene carrier, is the suppression of a specific intracellular pathway at mRNA level, essential for the survival of the cancer, which usually leads to apoptosis and the expression of proteins responsible for cell-cycle regulation or apoptosis.

To avoid the negative effects of using PAMAM as a carrier, it was also decided to modify their surface, core, and/or conjugate with other polymers, creating "hybrid vectors", as well as to create Supramolecular self-assembly nanocomplexes consisting of several dendrimers of different generations. The most common modification is pegylation, i.e., conjugation with polyethylene glycol. This reduces cytotoxicity and immunogenicity but comes at the expense of reduced tissue specificity and intracellular dendrimer uptake. In order to actively target the tissue, PAMAM dendrimers are also tethered, most commonly via PEG with amino acids, peptides, antibodies, hydrophobic particles, folic acid and various synthetic ligands (Table 1), which facilitate the precise delivery of the nanocarrier to the target site. This strategy has proven to be so successful that it has been widely adopted across multiple studies on PAMAM dendrimers, regardless of the complexity of the system. Overall, this is the most reasonable strategy to date, given the desire to achieve the highest possible efficacy and the minimization of cytotoxicity towards healthy cells, which is mainly possible by means of targeted delivery. 

Apart from surface modification with functional groups, an additional strategy often used in so-called hybrid vectors is the entrapment of gold nanoparticles between the inner dendrimer’s cavities, which increases their stability within the frame of the transfection environment, allowing for better therapeutic results. Aside from the aforementioned advantages of the most commonly used surface modification with functional groups, some of them can increase the dendrimer’s loading capacity for drugs and genetic material, such as the attachment of halloysite nanotubes.

One of the most interesting abovementioned nano-systems uses the HSV-TK/GCV-Cx43 system in the so-called suicide gene therapy. Suicide gene therapy is based on the so-called "bystander effect", which assumes that, to induce broad apoptosis in the cancer environment, only the small-cell fraction containing the suicide system is needed. This is a very promising strategy, especially when combined with PEG-modified dendrimers and a cellular-specific ligand, as it allows for the very precise and safe delivery of a given system to the cancer cells, in order to induce widespread apoptosis. Of course, there will always be a risk that the nano-complex will infiltrate into healthy cells, so the overall performance of a given complex will have to be evaluated in vitro and in vivo, using at least one primary and cancer line, in an appropriate animal model.

An equally innovative system used supramolecular self-assembly nanoparticles (SNP) composed of several PAMAM dendrimers of different generations in the formation of CAR-T cells expressing the EGFRvIII gene targeted towards hepatocarcinoma environment. CAR-T cell therapy creates great hopes in the fight against commercially non-curable types of cancer. A potential pathway for the development of non-viral nanocarriers, is harnessing their potential in CAR-T cell formation for glioblastoma multiforme therapies, which are of great clinical interest [165]. Despite the heterogeneous environment of glioblastoma, the EGFRvIII-dependent forms determine worse prognosis and survival in patients; therefore, this molecular target is of great therapeutic importance [166]. The way forward would be to re-evaluate EGFRvIII-CAR-T cells using other nano-systems that have a high potential for clinical and industrial up-scaling, which requires further research on suitable dendrimer-based nano-carriers for this task. Following this pathway, one can assume, with a higher probability compared to other PAMAM experimental systems, that this arrangement has a real chance of moving from the preclinical to the clinical phase. 

Moving away from the therapeutic use of dendrimers, one of the previously mentioned hybrid systems uses an anti-nucleolin aptamer (AS1411) attached to the surface. AS1411 is internalized upon binding to the appropriate receptor on the cancer cell surface and causes non-apoptotic cell death, called methuosis. Therefore, PAMAM dendrimers can also be used to study the mechanisms responsible for methuosis, using ligands such as AS1411.

The combination of cutting-edge technologies at the frontier of genetics and nanotechnology creates new opportunities and opens innovative development paths. One of these is the combination of CRISPR-Cas9 genome editing tool with dendrimer nanopolymers, which allows for the combination of the best features of the two worlds, namely safety, low cytotoxicity, high functional flexibility and effective endosomal escape, and unprecedented gene editing opportunities. The first reports of ground-breaking research have already been presented [167,168]. One of the studies used boronic-acid-rich G5 PAMAM dendrimer to deliver 13 proteins directly to the cell cytoplasm of HeLa cells, including the Cas9 ribonucleoprotein, essential for the recognition of CRISPR library elements [168]. PAMAMs decorated with phenylboronic acid show extraordinary protein delivery efficiency, one order of magnitude higher than naked PAMAM, and are about a dozen percent greater, compared to commercially available protein transduction reagents such as PULSin and TransEx. Equally importantly, the transported proteins could maintain their bioactivity after being delivered to the cytoplasm, where ribonucleoprotein Cas9 edited specific genome loci in 293T, HCT-116 and HT-29 cell lines. This demonstrates that PAMAM dendrimers with phenylboronic acid surface modification, conjugated with ribonucleoprotein Cas9, are an excellent tool for robust genome editing, not only for therapeutic properties, but also, in particular, for their biomedical purposes. More information on the remarkable CRISPR-Cas9 gene-editing technology is provided in [169,170].

**Figure 9:**
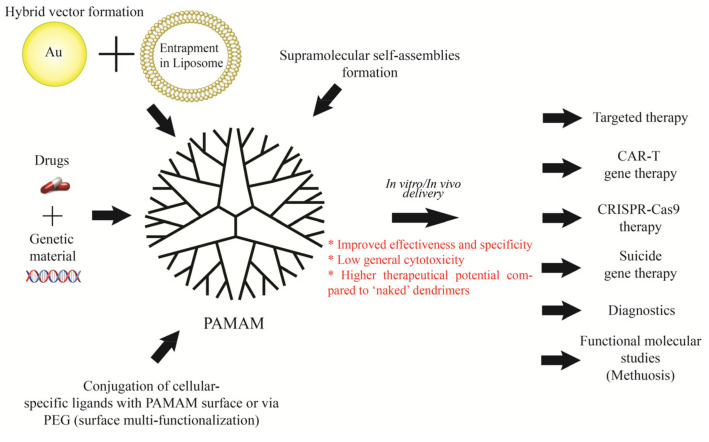
Modification approaches and practical applications of PAMAM dendrimers. Recent in vitro and in vivo proof-of-concept studies have demonstrated a multitude of new potential applications of PAMAM dendrimers by modifying their surface with a variety of cell-specific ligands, liposome encapsulation, and fixation of their spatial structure by immobilization of gold particles within internal branching cavities. These modification approaches seem to move the use of PAMAM dendrimers closer to anticancer targeted therapy involving commercially available chemotherapeutic agents, along with genetic material enhancing its therapeutic effect. The use of the PAMAM dendrimer also appears promising for CAR-T- and CRISPR-Cas9-based therapies. The use of PAMAM dendrimer also seems promising for CAR-T- and CRISPR-Cas9-based therapies as well as diagnostics and molecular functional studies.

A graphical summary of the above concepts is shown in Figure 9.

## 4. Future Prospects and Nanomedicine Regulatory Issues

Despite an increasing trend in the number of studies on non-viral carriers of genetic material and drugs, only a few have been introduced into clinical practice. Importantly, quite a substantial part of the research in the last two decades was associated with dendrimers, but only a few were translated into clinic and subsequently commercialized. It is quite striking that, despite the exponential growth in nanoscience articles and the enormous amount of work involved in the broadly defined development of nano-carriers, only a small fraction were approved in clinical trials, especially regarding dendrimers. However, it is noted that this trend is slowly changing and a growing amount of pre-clinical studies on dendrimers are being accepted by the clinical trial regulatory authorities.

Currently, the only clinical trials on dendrimers are being carried out by Starpharma, a company specializing in the research and development of dendrimers in the pharmaceutical and life science industries. Their leading invention, Dendrimer Enhanced Product^®^ (DEP^®^) is a drug delivery system which is being investigated in three parallel clinical trials. It is a platform based on poly-l-lysine dendrimers, decorated with a drug that is attached to the dendrimer surface by a PEG linker. DEP^®^ docetaxel, the docetaxel-delivery dendrimer-based conjugate, is currently in Phase II, where anticancer efficacy and safety will be tested on a group of 45 patients [171]. Clinical studies on systems with an analogous therapeutic approach, DEP^®^ cabazitaxel and DEP^®^ irinotecan are currently in the recruitment stage for Phase II [172,173]. Another application of the dendrimeric systems developed by Starpharma is VivaGel^®^, a microbicide for the prevention of HIV and HSV infections. 

Regarding clinical trials with poly-L-lysine dendrimers, an article was published in 2018 about the application dendrimer-based system called “ImDendrim” which aimed to transfer ^188^Rhenium- ligand (nitro-imidazole-methyl-1,2,3-triazol-methyl- di-[2-pycolyl] amine) for the treatment of colorectal cancer. An interventional study (NCT03255343) involving 10 patients with inoperable liver cancer resistant to classical chemotherapy showed that ImDendrim meets sufficient safety and efficiency standards [174]. Clinical trials are still ongoing.

Another challenge complicating the implementation of PAMAM dendrimers into clinical area is the “heat sterilization” phenomenon described by Krause et al [175]. The parenteral introduction of dendrimer-based therapeutics requires nanocomplex pre-sterilization. Krause et al. performed an experiment in which it was demonstrated that, upon heat sterilization at 134 °C at 2 bar for 25 min, PAMAM dendrimers did not retain their structure, whereas the polypropylenimine and polylysine dendrimers remained structurally stable. Sterilization by conventional sterile filters under aseptic conditions is a potential workaround for this problem, allowing for the effective elimination of microorganisms from the formulation [176]. Another important issue is the examination of the potential presence of endotoxins, following the sterilization process [177,178].

In recent years, intensive consultations have taken place between the scientific community and drug regulatory authorities in regions covered by their jurisdiction. In order to authorize dendrimer-based nano-systems for clinical trials by regulatory authorities, a comprehensive understanding of the pharmacokinetics (e.g., absorption, distribution, metabolism and excretion parameters) and pharmacodynamics of in-vivo-tested molecules is essential. Typically, three levels of nanoparticle development can be distinguished in terms of clinical translation, namely, (1) nano-pharmaceutical design, (2) preclinical evaluation and (3) clinical evaluation for commercialization. The issues in question have been highlighted and described in this article [179]. The requirements that the nanoparticle must meet in order to be approved for clinical settings are specified at each of these stages. In brief, some of the major challenges in this process are: (1) poorly standardized assay principles characterizing the quality of nanoparticles, and difficulties with large-scale production according to GMP standards; (2) shortage of dedicated toxicological assays for nanoparticles, the highly diverse structural stability of nanoparticles, insufficient understanding of intracellular interactions and limited accumulation in target tissues; (3) lack of explicit regulatory guidelines for nanoparticles, a complex patenting process and insufficient understanding of biological interactions in complex organisms.

As compared to biological and conventional drug molecules, dendrimers have far more complex and diverse physicochemical properties, resulting from the combination of additional characteristics, such as dendrimer generation, total binding surface, shape, type of core and branching, and the presence of functional groups on the surface (e.g., PEG, antibodies, inorganic molecules). In this respect, it constitutes one of the main factors hampering the establishment of clear and explicit guidelines for the authorization of nano-sized molecules for clinical practice. Wolfram J et al. claim that, in order to accept nano-sized molecules for clinical trials, it is sufficient to develop new tests and methodologies investigating cytotoxicity, without any major changes to the regulatory guidelines [180]. Therefore, a very promising opportunity, saving time and resources, would be the development of in vitro assays that accurately predict the in vivo toxicity of nano-sized molecules.

It is worth considering the formation of a bioinformatic database predicting the potential impact of dendrimers on cell physiology, depending on their physicochemical properties. Such a database would harmonize and systematize current knowledge on the influence of the above-mentioned parameters on cell physiology on different cell lines, hypothetically allowing for heuristic analysis of the influence of these parameters on internalization, distribution, dendrimer accumulation and cell metabolism. This would potentially reduce the time and resources needed for their characterization and the selection of appropriate methods to examine the most important parameters from a clinical context.

This concept, forming a foundation for the creation of such a database was created and accurately described by Donald A. Tomalia. Critical nanoscale design parameters (CNDPs) are the substantially enhanced conceptualization of the dendritic effect, which assumes that the physicochemical properties of dendrimers depend mostly on the generation of dendrimers, and, more precisely, their architecture and size, which is only part of a more complex system of relations. CNDPs describe the interaction between physicochemical, functional properties, and structural parameters such as shape, size, surface groups’ chemistry, flexibility/rigidity, architecture and elemental composition. Tomalia roughly qualifies CNDPs as "mini nano-periodic property patterns", indicating the possibility of ordering complex interdependencies in a logical, predictable and comprehensible manner, following the example of the Mendeelev table [181,182]. This would allow for the unification and standardization that is needed for the introduction of explicit rules allowing for the comparison of any type of dendritic-like nano-system, in terms of their physico-chemical properties. On this basis, it would also be possible to establish a new nano-framework to predict possible interactions of nano-systems with the cellular environment. The presence of such a generalized structural-functional framework will allow for as great a reduction in the number of variables used to describe nano-systems as possible, allowing for a better understanding of the dendrimer-related effects on cell physiology and a greater chance for translation into the clinical stage.

## 5. Concluding Remarks

Nanotechnology-related research is one of the most dynamically developing areas of science at present. The last 10 years of research have identified three leading modification strategies that might be useful from a pre-clinical and potentially clinical perspective, i.e., PAMAM with a multifunctional surface, hybrid vectors (macropolymer conjugates, liposomal entrapment, gold nanoparticles entrapment) and the formation of supramolecular self-assemblies. The most interesting systems with high therapeutic potential were those using the so-called "suicide gene approach", and utilizing CAR-T cells and the CRISPR-Cas9 gene-editing tool targeted towards the elimination of cancer cells. Despite the high degree of innovation, only a few dendritic systems have been successfully translated into clinical trials. Further efforts are needed to establish harmonized criteria and guidelines facilitating the seamless transition from the pre-clinical to the clinical phase, which will certainly contribute to greater innovativeness in nanomedicine in general. 

## Figures and Tables

**Table 1:** Overview of dendrimers with regard to their modifications and the type of delivered genetic material.

Dendrimer	Modification	Type of Modification	Genetic Material	Reference
PAMAM G4	Halloysite nanotubes	Hybrid vector, PAMAM grafted on halloysite nanotubes (engraftment on polymer)	siVEGF	Long et al. [68]
PAMAM G0	RGDfC peptide + PEG as a linker	Addition of RGDfC peptide PAM-ABP via PEG to PAMAM surface	pshVEGF	Kim et al. [89]; Nam et al. [90]
G5 PAMAM	Anti-HER2 nanobody (Nb)	Anti-HER2 Nb-conjugated with PAMAM	pGL4.14-pCXR1-tBi	Reshadmanesh et al. [91]
G5 PAMAM	Anti-EGFR h-R3 antibody on the PAMAM surface	Addition of antibody to PAMAM surface	siMDR1	Li et al. [93]
PAMAM G5	h-R3 antibody + human serum albumin (HSA) + EGF	Addition of h-R3 antibody + human serum albumin (HSA) + EGF to PAMAM surface	siPLK1	Li et al. [97]
PAMAM G5	PEG + lactobionic acid (Gal)	Addition of PEG + Gal to PAMAM surface	AEG-1 siRNA + all-trans retinoic acid (ATRA)	Rajasekaran et al. [98]
PAMAM G5	EDC + Folic acid	Addition of folic acid to modified PAMAM surface (via EDC)	ODNs EGFR	Kang et al. [108]
PAMAM G4	Histidine, arginine and lysine	Addition of histidine and arginine or histidine and lysine to PAMAM surface	Plasmid with apoptin gene	Bae et al. [110]
PAMAM	Folic acid	Addition of folic acid to PAMAM surface	miR-7	Liu et al. [111]
PAMAM G5	PEG + angiopep-2	Addition of PEG + angiopep-2 to PAMAM surface	pORF-TRAIL	Huang et al. [112]
PAMAM G5	PEG-transferrin	Addition of PEG-transferrin to PAMAM surface	pORF-hTRAIL	Gao et al. [115]; Huang et al. [116]
PAMAM G4	Folic acid	Addition of folic acid to PAMAM surface	siVEGFA	Xu et al. [122,123]
PAMAM G5	catechol-PEG and catechol-PEG- RGD	Addition of catechol-PEG and catechol-PEG-RGD to PAMAM surface modified with phenylboronic acid (PBA)	siPLK1	Liu et al. [158]
PAMAM G5	PEG + gold nanoparticles (AuNP) + Gemcitabine	Hybrid vector, addition of PEG, miR-21 and Gemcitabine to PAMAM surface and AuNP entrapment	miR-21 inhibitor	Lin et al. [138]
PAMAM G5	CBAA + gold nanoparticles	Hybrid vector, addition of carboxybetaine acrylamide (CBAA) to PAMAM surface and AuNP entrapment	Plasmid with hypermethylated in cancer 1 (HIC1) gene	Xiong et al. [159]
PAMAM G4	Carbon nanohorns (CNHs)	Hybrid vector, PAMAM anchored to CNHs (f-CNH3)	siRNA against cofilin-1	Pérez-Martínez et al. [139]
PAMAM G4	PEG + 10-bromodecanoic acid (10C) and AS1411 aptamer	Hybrid vector, 10C-PEG attached to PAMAM surface and conjugation with AS1411 aptamer via PEG	shRNA-Bcl-xL	Ayatollahi et al. [141]
PAMAM G5	PEG + EpDT3 aptamer	Hybrid vector, PEG attached to PAMAM surface and conjugation with EpDT3 aptamer via PEG	plasmid-encoding tumor suppressor lncRNA MEG3 (pMEG3)	Tai et al. [160]
PAMAM G5	Phospholipid groups on the PAMAM surface	Hybrid vector, phospholipid with PAMAM	siMDR1	Liu et al. [145]
PAMAM G1	CD-PEI10000, Ad-PAMAM, and Ad-PEG modified with- GE11 and GALA	Supramolecular self-assembled nanoparticles (SNPs)	shVEGF	Lu et al. [152]
Adamantane-grafted PAMAM G1-G5	Adamantane-grafted PEG (Ad-PEG), cyclodextrin-grafted branched PEI25000 (CD-PEI25000)	Supramolecular self-assembled nanoparticles	pcDNA3.1(+) plasmid containing anti-EGFRvIII scFv-CD28-4-1BB-CD3ζ expression cassette (T-CAR therapy)	Yu et al. [153]
PAMAM G5	G3 PAMAM	Supramolecular vector, addition of G3 PAMAM to G5 PAMAM via β-cyclodextrin	miR-21 inhibitor	Song et al. [157]
